# Causal Composition: Structural Differences among Dynamically Equivalent Systems

**DOI:** 10.3390/e21100989

**Published:** 2019-10-11

**Authors:** Larissa Albantakis, Giulio Tononi

**Affiliations:** Department of Psychiatry, Wisconsin Institute for Sleep and Consciousness, University of Wisconsin-Madison, Madison, WI 53719, USA

**Keywords:** integrated information, causation, graphical models, organizational structure, multivariate interaction, agency

## Abstract

The dynamical evolution of a system of interacting elements can be predicted in terms of its elementary constituents and their interactions, or in terms of the system’s global state transitions. For this reason, systems with equivalent global dynamics are often taken to be equivalent for all relevant purposes. Nevertheless, such systems may still vary in their causal composition—the way mechanisms within the system specify causes and effects over different subsets of system elements. We demonstrate this point based on a set of small discrete dynamical systems with reversible dynamics that cycle through all their possible states. Our analysis elucidates the role of composition within the formal framework of integrated information theory. We show that the global dynamical and information-theoretic capacities of reversible systems can be maximal even though they may differ, quantitatively and qualitatively, in the information that their various subsets specify about each other (intrinsic information). This can be the case even for a system and its time-reversed equivalent. Due to differences in their causal composition, two systems with equivalent global dynamics may still differ in their capacity for autonomy, agency, and phenomenology.

## 1. Introduction

**Figure 1:**
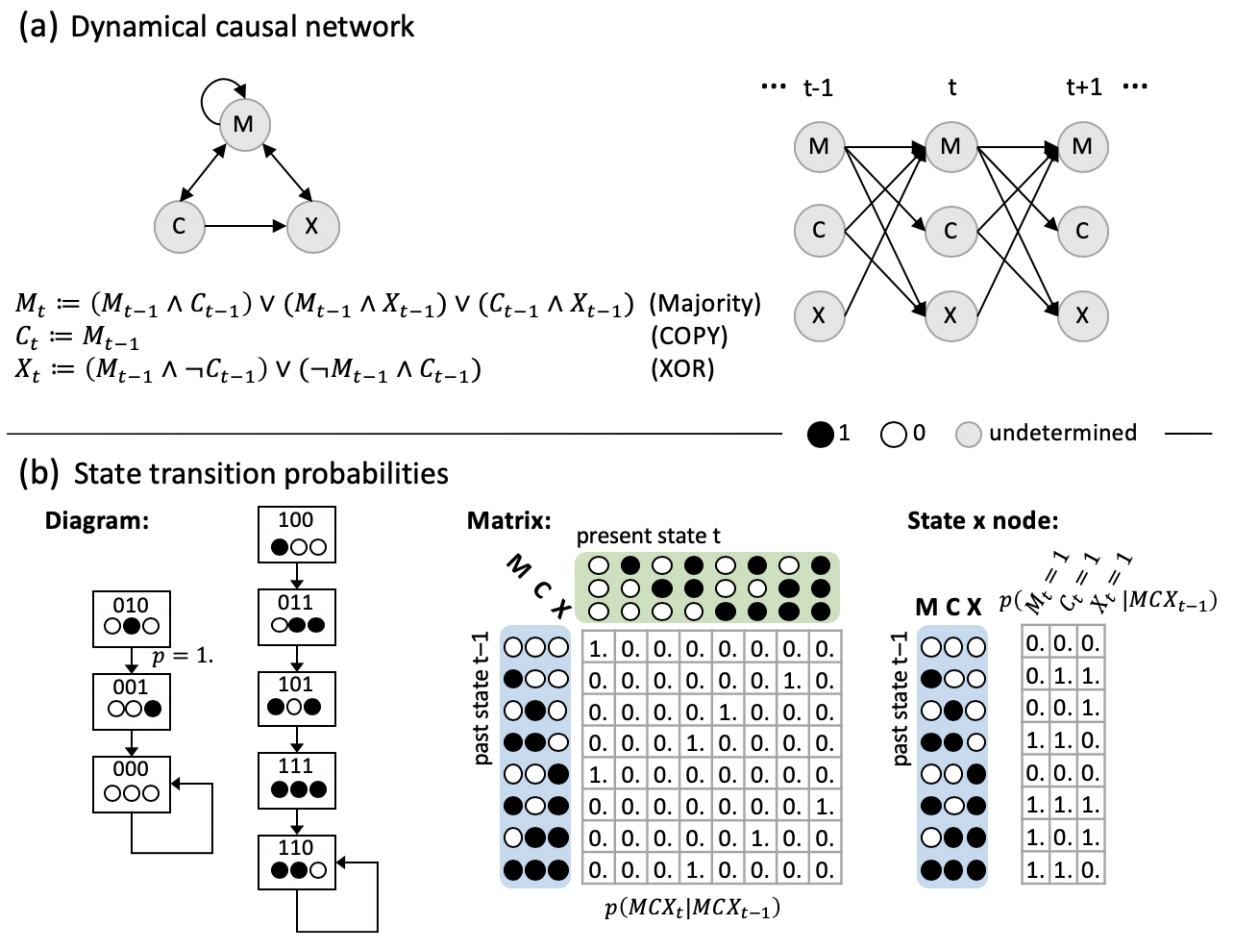
An example neural network of three binary interacting elements. The system evolves in discrete time steps and fulfills the Markov property, which means that the conditional probability distribution of the system at time *t* depends only upon its prior state at t−1. Shown are two equivalent descriptions of the system, which allow us to model and predict its dynamical state evolution: (**a**) The system represented as a dynamical causal network. This type of description corresponds to a reductionist view of the system, highlighting the interactions between individual elements. Edges indicate causal connections between elements, which are equipped with update functions, or structural equations, that specify the element’s output given a particular input. While the neural network (left) is recurrent, it can be represented by a directed acyclic graph (DAG) when unfolded in time (right). Throughout, we assume stationarity, which means that the system’s dynamics do not change over time. (**b**) The system represented by its state transition probabilities under all possible initial conditions, illustrated in form of a state transition diagram (left), and transition probability matrix (middle). This type of description corresponds to a holistic perspective onto the system, taking the system states and their evolution in state space as primary. As the system elements are binary (and comply with Equation (2), Section 5.1), the transition probability matrix can also be represented in state-by-node format, which indicates the probability of each node to be in state ’1’ at *t* given the respective input state at t−1 (right). As the system is deterministic, all probabilities are either 0.0 or 1.0. To distinguish binary state labels from real-valued probabilities, the latter include decimal points.

Traditionally, how well we can predict the behavior of a system is taken as a measure of how well we are able to model, and thus “understand”, the system [1] (but see [2,3,4]). In our view, approaches to capture and model a system’s dynamics can be roughly divided into reductionist approaches that model how the system’s elementary constituents update and interact, and holistic approaches that model the dynamical evolution of the system as a whole based on its state transition probabilities (Figure 1). Predicting a system’s dynamics therefore does not require explicit knowledge about the system’s causal composition, that is, in which way the various subsets of elements (mechanisms) within the system interact and what information they specify about each other.

On the other hand, characterizing the functional role of particular parts of a system and the way in which they interact has always been a main line of inquiry in the sciences dealing with complex systems, such as biology and, most prominently, neuroscience [5,6,7]. In neuroscience, information theoretical approaches [8,9,10,11,12,13] are utilized to identify the presence of information about some external variable or stimulus in a specific part of the system. This part is then said to “represent” the variable or stimulus as its informational content [14,15,16,17] (but see [18] for a critical discussion). More recently, machine-learning based techniques such as “decoding” have gained popularity [11,17,19] and have been utilized to investigate content-specific neural correlates of consciousness [20,21]. While these approaches recognize that subsets within the system may carry out different functional roles, they consider correlations between objects or events from an extrinsic point of view rather than the causal consequences within the system [10,20,22,23]. Moreover, the focus is put on prediction, rather than understanding [3].

Originally conceived as a theory of consciousness [24,25,26], integrated information theory (IIT) provides a theoretical framework intended to characterize a system’s intrinsic information, the information that a system, in its current state, specifies about itself [27]. By contrast to the standard, information-theoretic notion of information (“Shannon information”), intrinsic information in IIT is state-dependent and causal in nature [27,28]. Moreover, information in IIT is *compositional*: the various subsets within a system may specify irreducible information about each other.

In this way, the formal framework of IIT offers the tools to address several issues related to *autonomy* and *agency*, with respect to which, measures that are primarily aimed at predicting a system’s dynamical evolution in holistic or reductionist terms generally fall short. This includes questions regarding actual causation (“what caused what?”) [29,30,31], how to identify individuals and their causal borders [27,32,33,34,35], and how to characterize the compositional nature of phenomenal experiences [27].

In a complementary contribution to this special issue [31], we demonstrated how the causal principles of integrated information theory, including composition, apply in the context of actual causation, where the objective is to assess “what caused what” within a transition between subsequent states of a discrete, distributed dynamical system.

Here we focus on the role of composition in characterizing the intrinsic information of a system of interacting elements. Our goal is to highlight the importance of composition for understanding complex systems, such as neural networks. For the purpose of this paper, we will ignore other aspects of the mathematical framework of IIT, such as the exclusion postulate and the choice of an appropriate intrinsic distance measure [27]. To this end, we first describe a simplified account of intrinsic information—the information that a system specifies about itself—which is largely based on standard information-theoretical measures. Next, we analyze composition in a random sample of 10,000 deterministic and probabilistic, binary 3-node systems, as well as the full set of all reversible, binary 3-node systems (totaling 40,320). Within this sample, we especially focus on the class of ergodic, reversible (ER) systems (see Section 5.6), which cycle through all their possible states and thus specify the same, maximal amount of effective [36,37] and predictive information [38] (3 bits). We demonstrate that the causal composition, intrinsic information, and integration of these systems may still vary, revealing structural properties that cannot be captured in reductionist or holistic terms. Notably, even pairs of systems whose dynamics are identical under time-reversal are typically composed of an entirely different set of mechanistic components, and may thus differ in their amount of intrinsic and integrated information.

Finally, we will discuss: (1) Differences and similarities between the notion of composition portrayed in this work and accounts of information decomposition [39,40,41,42], (2) the role of composition with respect to a system’s capacity for autonomy and agency, and (3) the role of composition within IIT as a theory of phenomenal consciousness.

## 2. Theory

To start, we consider a system *S* of three interacting, binary elements (“bits”), as shown in Figure 1. The maximum uncertainty, or “Shannon” entropy *H*, of this system is 3 bits, as there are eight possible states. Being able to predict the next state of such a system also amounts to maximally 3 bits of “Shannon” information (corresponding to the reduction of uncertainty if all eight system states are equally likely a priori). The mutual information between the previous and present states of the set of system variables $V_{t-1}$ = $V_{t}$ = *S*, $I\left(V_{t-1};V_{t}\right)=H\left(V_{t}\right)-H\left(V_{t}|V_{t-1}\right)$ (see Equation (3), Section 5.2), has been termed *predictive information* [38]. It measures the average amount of information that a state $V_{t-1}=v_{t-1}$ specifies about the next state $V_{t}=v_{t}$, and vice versa. Imposing a uniform distribution on the states of $V_{t-1}$, we obtain the *effective information* (Equation (5)) [36,37], a holistic measure of causal information, which is 2.5 bits in our example system.

### 2.1. The Compositional Intrinsic Information of an Example System

Here we are interested in the intrinsic information that a system in its current state specifies about its intrinsic causes (its prior state) and effects (its next state). MCX is constituted of three binary elements, each equipped with its own input-output function, which can be interpreted as a mechanism to infer information about MCX’s prior state. *M*, for example, implements a majority function, turning on (‘1’) whenever at least two elements of MCX were on at t−1. We will consider MCX=(0,1,1) as our example state in the following. Given that $M_{t}=0$, the system MCX had to be in one out of four possible states at t−1, namely those with |MCX|<2. $M_{t}=0$ thus reduces the uncertainty about the state of MCX at t−1. Likewise, $C_{t}$ copies the state of $M_{t-1}$, and thus evaluates the question “Was $M_{t-1}=1{?}^{\prime \prime }$. Being in state $C_{t}=1$, it specifies that $M_{t-1}$ must have been on (‘1’). We will consider first the requirements for intrinsicality, then composition, then integration.

**Figure 2:**
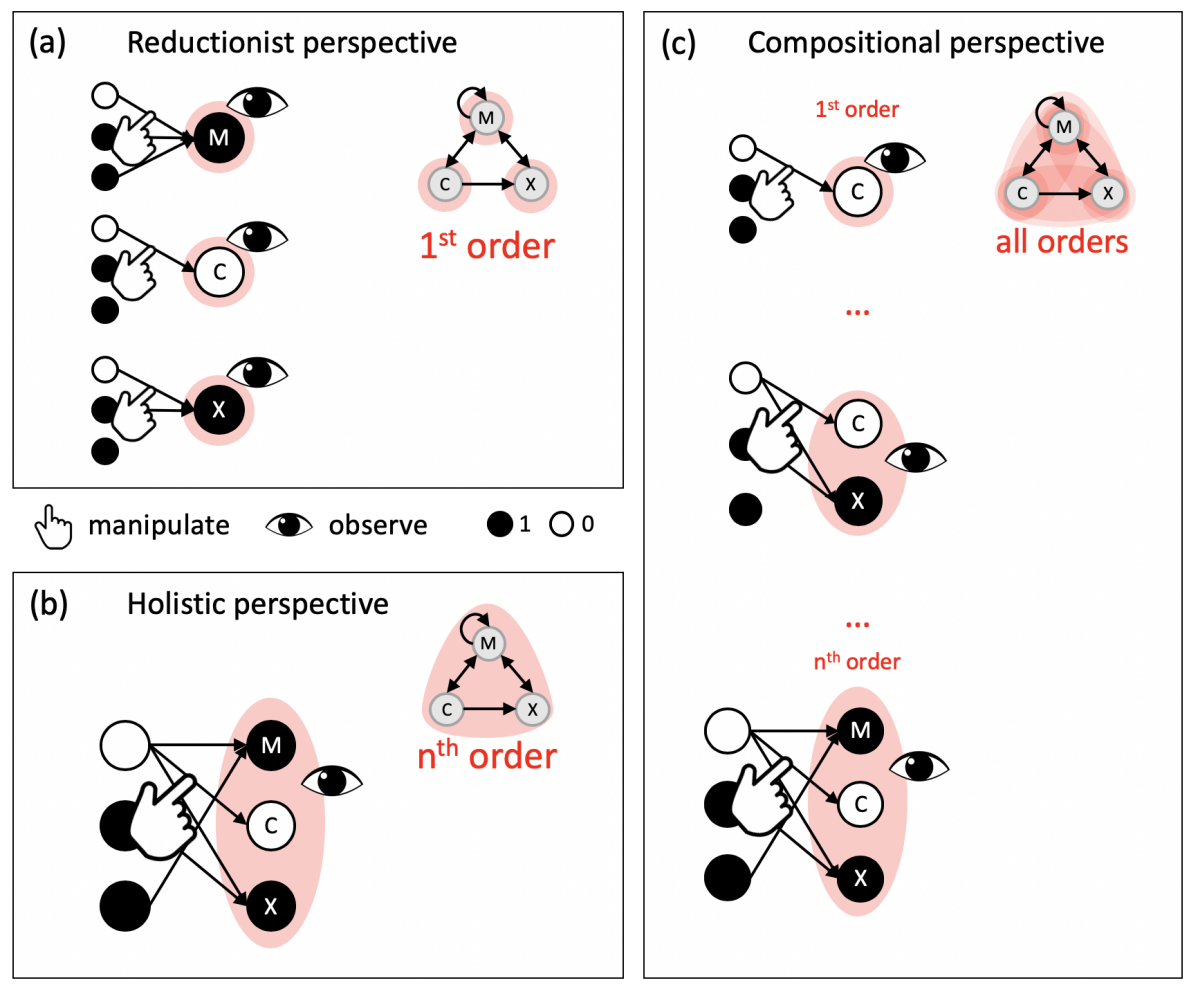
Reductionist, holistic, and compositional perspectives. (**a**) From a reductionist perspective, causal interactions are evaluated at the level of individual elements (first order). Once the state of the individual elements is observed, the state of the system and all its subsets have to be inferred. (**b**) Taking a holistic perspective, causal interactions are evaluated at the global level of the entire system ($n^{th}$ order). Once the global state is observed, the states of all system subsets have to be inferred. (**c**) From a compositional perspective, causal interactions are evaluated at all orders. Information about the state of each subset is available in explicit form if it is specified (irreducibly) by another subset within the system.

**Intrinsicality:** From an extrinsic perspective, the entropy *H* of a system is also a lower bound on the expected number of “yes/no” questions needed to determine the system’s state [43]. This implies that once the state of every single unit is known, so is the state of all the units together and all its subsets. Conversely, once the state of all the units is known, so is the state of every single unit and all their combinations (Figure 2). Providing this information in addition would seem redundant as it can easily be inferred. However, information that has to be inferred remains implicit. To make it explicit, a function (mechanism) has to be applied. From the intrinsic perspective of the system, information about its causes and effects is thus only available if it is made explicit by some mechanism within the system. In other words, the system itself takes a compositional perspective (Figure 2).

**Composition:** While the reductionist and holistic perspectives focus on causal interactions at one particular order (single elements vs. the system as a whole), any set of elements within the system that receives inputs from and provides outputs to elements within the system may, in principle, form a separate mechanism within the system (Figure 2). Any set of elements within the system may thus specify its own intrinsic information about the prior (and next) state of a particular system subset—its cause (or effect) “purview”. The constraints that a set of system elements in a state specifies about the prior state of a system subset are captured by its *cause repertoire* (Equation (9), Section 5.3).

**Figure 3:**
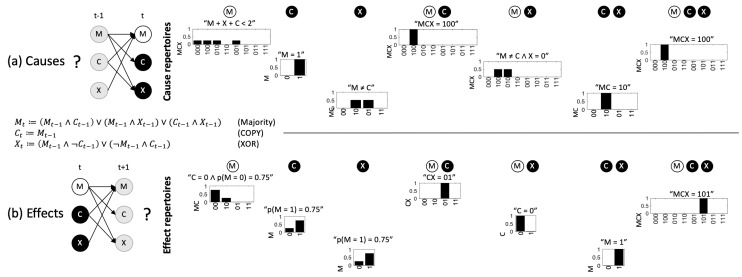
Cause and effect repertoires of example system MCX in state (0,1,1). The cause (effect) repertoires of individual system elements and their combinations specify how each set of elements in its current state constrains its possible causes (effects) within MCX. $C_{t}=1$, for example, specifies that $M_{t-1}=1$, and predicts that $M_{t+1}=1$ is likely with p=0.75. Labels above the repertoires indicate what each set of elements specifies about its “purviews” (see Section 5.4), the system subsets that are being constrained, which also determine the size (state space) of the repertoire in the figure. $C_{t}=1$, for example, does not constrain $C_{t+1}$ or $X_{t+1}$ in any way. Given $C_{t}=1$ the state of $C_{t+1}$ and $X_{t+1}$ remains maximally uncertain.

The cause repertoire illustrates the potential causes for the set of elements to be in its particular state at time *t* within the system, assuming no other knowledge but its mechanistic structure. As shown in Figure 3, in system MCX, $MC_{t}=\left(0,1\right)$, for example, specifies that the previous system state must have been $MCX_{t-1}=\left(1,0,0\right)$, and $CX_{t}=\left(1,1\right)$ specifies that $MC_{t-1}=\left(1,0\right)$, since $CX_{t}=\left(1,1\right)$ whenever $MC_{t-1}=\left(1,0\right)$, and not in other cases. Even in a deterministic system, the previous state of a subset may not always be perfectly specified. For example, $M_{t}=0$ specifies that the system’s elements at t−1 were more likely to be ‘0’ than ‘1’ (their sum being smaller than 2).

While $MC_{t}=\left(0,1\right)$ here determines the system’s prior state completely, there is no set of elements within the system that explicitly specifies any information about the state of $C_{t-1}$ and only $C_{t-1}$. The information that $C_{t-1}=0$, while contained in $MC_{t-1}=\left(1,0\right)$ as specified by $CX_{t}=11$, remains implicit and thus extrinsic. Without a mechanism that explicitly specifies the previous state of *C* and only *C*, from the intrinsic perspective, the system itself cannot perform the necessary inference. In short, composition reveals all the mechanisms within a system and the information they specify about the system’s intrinsic causes and effects.

In the same way that the sets of elements within MCX may specify information about the prior state of various system subsets, they may also specify information about the next state of particular subsets (Figure 3, bottom). The potential effects of each set within the system are illustrated by its *effect repertoire* (Equation (8), Section 5.3). Since the next state of a particular system element may depend on the state of multiple elements at time *t*, the predictions of system subsets may again be probabilistic even in a deterministic system. $C_{t}=1$, for example, only specifies that $M_{t+1}$ is more likely to be on than off with $p(M_{t+1}=1)=0.75$, assuming that the state of the other inputs to $M_{t+1}$ (and the other elements) is maximally uncertain and independent. For the same reason, two subsets may specify contradictory predictions. $M_{t}=0$, for example, entails that $M_{t+1}$ is more likely to be off, contrary to the predictions of $C_{t}$ and $X_{t}$, which specify that $M_{t+1}$ is more likely to be on.

**Integration:** Next, we must assess whether and to what extent a set of elements specifies *irreducible* information about other system subsets. This is because a set of elements contributes to the intrinsic information of the system as a whole only to the extent that it is irreducible under any partition (see Section 5.4, Equation (13)). This is quantified by its irreducible information $\varphi_{C/E}$, which measures the minimal difference (here using $D_{KL}$) between the cause/effect repertoire before and after a partition, evaluated across all possible partitions (Equation (15)). In principle, each of the $2^{3}-1=7$ subsets of the system could specify irreducible information about the prior and next state of different subsets within MCX, and thus contribute to the system’s intrinsic information in a compositional manner. In our example system, the information specified by the “third-order” set $MCX_{t}=\left(0,1,1\right)$, however, is identical to the information specified by its subset $MC_{t}=\left(0,1\right)$. The information that $MCX_{t}=\left(0,1,1\right)$ specifies about $MCX_{t-1}$ is only due to $MC_{t}=\left(0,1\right)$. Including $X_{t}=1$ does not contribute anything on top; it can be partitioned away without a loss of information. Similarly, $MX_{t}=\left(0,1\right)$ does not specify irreducible information, since the information that $C_{t+1}=0$ is due to $M_{t}=0$ alone. The irreducible information specified by the subsets in our example system $MCX_{t}$ in state (0,1,1) are listed in Table 1. In the following we will quantify the total amount of intrinsic information specified by a particular system as $\sum \varphi_{C}+\sum \varphi_{E}$, which is 8.81 bits for $MCX_{t}=\left(0,1,1\right)$.

### 2.2. Causal Composition and System-Level Integration

While we can characterize the causal composition and $\sum \varphi_{C}+\sum \varphi_{E}$ of any set of elements, the notion of “intrinsic information” really only makes sense if there *is* a system in the first place, meaning one “whole” as opposed to multiple separate sets [23,27]. To establish whether a (sub)set of elements forms a whole in an observer-independent manner, purely reductionist or holistic approaches are inadequate [33]. Within the IIT framework, a set of elements can only form a whole if all of its parts contribute irreducible information about the prior and next state of the rest. This is quantified by Φ (“big phi”), the system-level integrated information [27]. To measure Φ the system is partitioned and the amount of intrinsic information lost due to the partition is quantified, just as for φ. For Φ, this means that we evaluate how the partition affects the intrinsic information specified by all of the system’s subsets.

Here we define a simplified measure of Φ, termed $\Phi_{\subseteq }$ (“compositional big phi”, indicated by the ⊆ symbol), which takes the causal composition of a set of elements *S* into account. The measure $\Phi_{\subseteq }$ omits several other aspects of the canonical measure [27], which evaluates all requirements that IIT poses for a physical substrate of consciousness. Specifically, for $V_{t}=S$ in state $v_{t}$:

(1) $$\Phi_{\subseteq }\left(v_{t}\right)=\min_{\Psi }\left(\min\left(\sum \Delta \varphi_{C}\left(v_{t}\right),\sum \Delta \varphi_{E}\left(v_{t}\right)\right)\right),$$

where $\Delta \varphi_{C/E}$ denotes the difference in $\varphi_{C/E}$ before and after a system partition Ψ, over which the measure is minimized (see Section 5.5 for details). Taking the minimum between the cause and effect side corresponds to the notion that the system in its present state acts as an “information bottleneck” and guarantees that a system with $\Phi_{\subseteq }>0$ specifies irreducible information about the prior and next state of its subsets [27]. The system MCX in state (0,1,1) specifies a value of $\Phi_{\subseteq }=1.02$ bits, where the minimum is found for $\sum \Delta \varphi_{E}$, under a partition that renders the elements MC at t+1 independent of *X* at *t*. This eliminates the information specified by $X_{t}=1$, $CX_{t}=\left(1,1\right)$, and $MCX_{t}=\left(0,1,1\right)$ about their respective purviews in $MCX_{t+1}$.

## 3. Results

**Figure 4:**
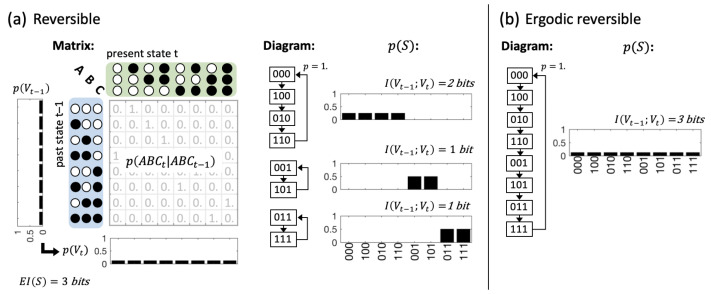
Informational and dynamical properties of reversible and ergodic-reversible (ER) discrete dynamical systems. (**a**) An example of a reversible three element system S={A,B,C}. EI(S)=n bit for all reversible systems. Dynamically these systems can still specify between 1 and $2^{n}$ attractors that lead to different stationary distributions p(S) depending on the initial state; (**b**) example of an ergodic reversible (ER) system. In these systems, $I\left(V_{t-1};V_{t}\right)≃EI\left(S\right)=n$ bit as the system cycles through all of its possible states, and the observed, stationary distribution p(S) converges to a uniform distribution for an infinite number of observations and every full cycle through the system’s state space.

To investigate variations in causal composition and integration between systems with equivalent global dynamics, we consider the data set of reversible, binary 3-node systems, and, within those, the subset of ergodic-reversible (ER) systems (Figure 4, Methods Section 5.6). Reversible systems may still exhibit multiple fixed points and/or periodic cycles, and thus display different stationary distributions depending on their initial state. By contrast, ER systems cycle through all their possible states, which leads to a uniform stationary distribution of system states. EI(S)=3 bits and $\langle H\left(V_{i,t+1}\right)\rangle =1$ bit for all reversible systems. In ER systems, the predictive information $I(V_{t-1};V_{t})$ (Equation (3)), which is typically based on observed distributions converges to EI(S). Focusing on ER systems thus has the additional advantage that we can set aside reservations about imposing a uniform distribution for $V_{t-1}$, as the stationary, observed distribution in these systems is the uniform distribution. This also means that the TPM of an ER system, and all subsequently computed quantities, can be derived from observation [27,44].

For comparison, we also evaluate two sets of 10,000 random 3-node systems, one deterministic, the other probabilistic. As shown in Appendix A, $\sum \varphi_{C}+\sum \varphi_{E}$, the total (compositional) amount of intrinsic information specified by a system, is strongly correlated with the system’s effective information EI(S) in these comparison data sets. Our goal in the following, however, is to highlight the remaining variance in $\sum \varphi_{C}+\sum \varphi_{E}$ and $\Phi_{\subseteq }$ once the informational and dynamical properties at the highest level are fixed.

### 3.1. Same Global Dynamics Different Composition and Integration

**Figure 5:**
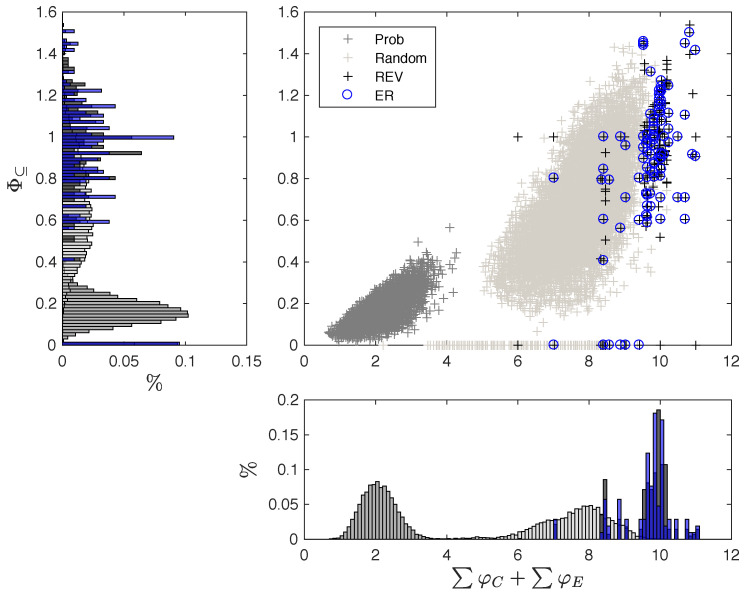
Distribution of intrinsic information and system-level integrated information. $\Phi_{\subseteq }$ is plotted against $\sum \varphi_{C}+\sum \varphi_{E}$ for all evaluated data sets: a random sample of 10,000 probabilistic (“Prob”) and deterministic (“Random”) TPMs, as well as the set of all 40,320 reversible systems (“REV”), and the subset of 5040 ergodic reversible (“ER”) systems (see Section 5.6 for details). $\Phi_{\subseteq }$ and $\sum \varphi_{C}+\sum \varphi_{E}$ are averages across all possible system states. Histograms show the distribution of $\Phi_{\subseteq }$ values (left) and $\sum \varphi_{C}+\sum \varphi_{E}$ values (bottom).

Figure 5 shows the relation between average $\Phi_{\subseteq }$ and $\sum \varphi_{C}+\sum \varphi_{E}$ for each evaluated data set. Higher values of $\sum \varphi_{C}+\sum \varphi_{E}$ allow for larger $\Phi_{\subseteq }$ values. This is because more intrinsic information may be lost due to a system partition. Nevertheless, even systems with high $\sum \varphi_{C}+\sum \varphi_{E}$ may not be integrated ($\Phi_{\subseteq }=0$). Probabilistic systems generally have smaller values of $\sum \varphi_{C}+\sum \varphi_{E}$, and thus less capacity for $\Phi_{\subseteq }$, since their elements, alone and in combination, specify less information due to noise.

**Figure 6:**
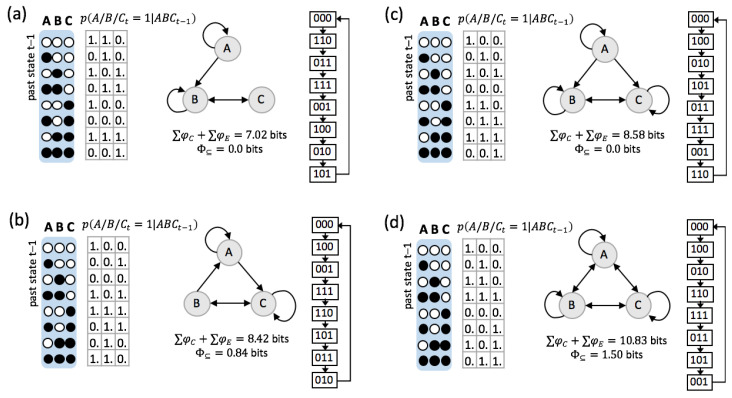
Illustrative ER example systems from low to high $\sum \varphi_{C}+\sum \varphi_{E}$. (**a**) An ER system with the lowest $\sum \varphi_{C}+\sum \varphi_{E}$. Nodes *A* and *C* are both simple NOT/COPY logic gates. *A* is only connected to *B* in a feedforward manner, thus $\Phi_{\subseteq }=0$. (**b**) An ER system with slightly higher $\sum \varphi_{C/E}$ than (a). *B* is a simple COPY logic-gate, *A* is an XOR. This system is integrated with $\Phi_{\subseteq }=0.84$. (**c**) An ER system with higher $\sum \varphi_{C}+\sum \varphi_{E}$, but $\Phi_{\subseteq }=0$. *A* is a simple NOT logic-gate (same as in (a)) that connects to *B* and *C* in a feedforward manner. (**d**) An ER system with high $\sum \varphi_{C}+\sum \varphi_{E}$. All nodes specify nonlinear input-output functions over all system elements and the system is strongly integrated with $\Phi_{\subseteq }=1.50$.

While reversible systems typically have high values of $\Phi_{\subseteq }$ and $\sum \varphi_{C}+\sum \varphi_{E}$ compared to the random sample of deterministic systems, some are still reducible with $\Phi_{\subseteq }=0$, as also exemplified below in Figure 6a,c. Finally, in terms of their composition and integration, the subset of ER systems does not differ significantly from the set of all reversible systems.

In Figure 6 we take a closer look at four examples across the range of possible ER systems. As the examples demonstrate, “perfect” dynamics and predictability ($I(V_{t-1};V_{t})=3$ bits) can be implemented by systems composed of various elementary mechanisms with qualitatively different connection patterns.

Taking a reductionist perspective, greater composition is associated with more complex elementary mechanisms (nonlinear functions across multiple inputs). Taking a holistic perspective, this corresponds to a more distributed implementation of the computation within the system. Nevertheless, only a compositional analysis that takes all intermediate levels into account can provide a complete picture of the system’s causal and informational structure, which is necessary to understand how the individual elements interact and compose joint causal constraints.

As we have argued in Section 2.1, $\sum \varphi_{C}+\sum \varphi_{E}$ quantifies the intrinsic information that the various subsets within a system in their current state specify about each other’s prior and next states. Table 2 lists the compositional information of the four example systems in Figure 6 for one particular example state ($ABC_{t}=\left(0,1,1\right)$).

All ER systems share equivalent global dynamics, as they cycle through all their possible states. (Note that from a holistic perspective only the state transition diagram matters, not the individual state labels.) For this reason, also their predictive and effective information are maximal. Nevertheless, they still differ in how much and which information the systems specify about themselves from a compositional perspective (Figure 2). In Table 3, for example, we compare the two systems shown in Figure 6a,d in terms of the predictions that each of their irreducible system subsets makes about the next state of other subsets within the system. Both systems, at the highest order ($ABC_{t}=\left(0,1,1\right)$), specify (predict) the next state of the system as a whole. From an extrinsic perspective, it would thus be easy to infer the next state of each individual system element. However, such an inference requires an additional mechanism to read out this information. Within system (d) (Table 3, right), each of the second order subsets correctly specifies the next state of a different system element. Within system (a), only $A_{t+1}=1$ and $C_{t+1}=1$ are correctly specified.

### 3.2. Global vs. Physical Reversibility

As demonstrated above, dynamically reversible systems as defined here may vary with respect to their irreducibility ($\Phi_{\subseteq }$) and the intrinsic information they specify, even though from a holistic perspective they all specify the same dynamics.

**Figure 7:**
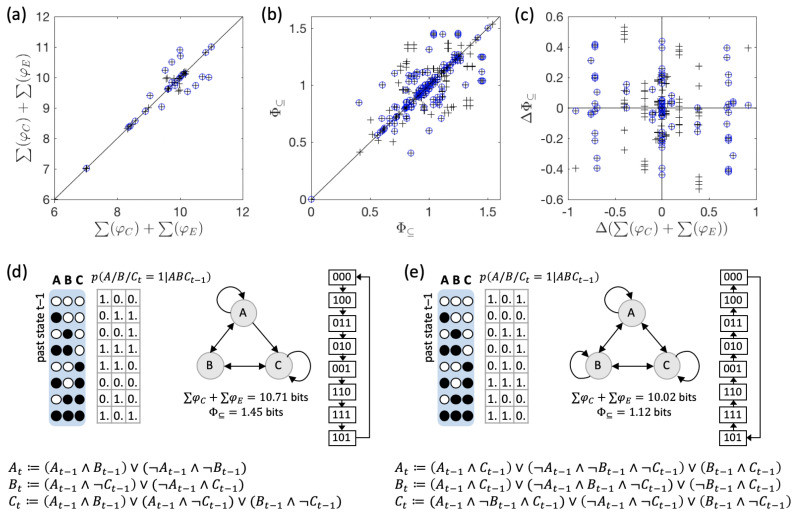
Intrinsic information and system irreducibility under time-reversed dynamics. (**a**,**b**) The total amount of intrinsic information $\sum \varphi_{C}+\sum \varphi_{E}$ (a) and $\Phi_{\subseteq }$ (b) of each system is plotted against its time-reversed dynamical equivalent, which can exhibit different values. (**c**) The difference in $\Phi_{\subseteq }$ between a system and its reverse, plotted against their difference in $\sum \varphi_{C}+\sum \varphi_{E}$. (**d**) Example of a system with different causal composition and $\Phi_{\subseteq }$ compared to its time-reversed dynamical equivalent shown in (**e**). Note also the differences in their elementary mechanisms and connectivity. Compared to (e), in (d) node *B* lacks the self-connection and *A* does not receive an input from *C*. While node *A* in (d) implements biconditional logic and node *B* an XOR function, all nodes in (e) implement logic functions that depend on *A*, *B*, and *C* as inputs.

As a final point, we compare each reversible system in our data set with its time-reversed dynamical equivalent. The results are shown in Figure 7. While some system pairs do specify the same amount of $\sum \varphi_{C}+\sum \varphi_{E}$ and $\Phi_{\subseteq }$, more than half of all pairs differ in either or both of these quantities. The example pair of systems shown in Figure 7d,e, moreover, demonstrates that a system and its complement under time-reversal may differ in their elementary causal dependencies (connectivity diagram), basically specifying two completely different systems in terms of their mechanistic organization.

As defined in Section 5.6, reversibility refers to the global dynamics of a discrete dynamical system with a finite state space. Such global reversibility does not imply local reversibility. This means that the elementary mechanisms that constitute the system are not typically reversible. For elements with one binary output, all input-output functions except for COPY and NOT logic-gates are necessarily convergent (multiple inputs may lead to the same output) and thus logically irreversible (see also [45] for a recent review on reversible cellular automata).

Reversibility (in particular dynamical reversibility), is often associated with the notion of being able to “run the system in reverse”. However, systems whose dynamics are globally but not locally reversible do not comply with this notion. As our results highlight, implementing the reversed dynamics would require different physical mechanisms than those of the original system. The direction in which the system evolves is thus determined by its underlying mechanisms and cannot actually be reversed. This shows that global dynamical equivalence does not imply physical equivalence in a more general sense.

## 4. Discussion

In this study we have explored the notion of causal composition in small, discrete dynamical systems, with a specific focus on a data set of “ergodic reversible” systems that display the same global dynamics as they cycle through all their possible states. These systems are characterized by a maximal amount of predictive and effective information. Nevertheless, they may vary in the intrinsic information specified by their various subsets. As argued above, from the intrinsic perspective of the system itself, the only information that is available to the system is information that is made explicit by the system’s mechanisms. Such information is necessarily causal, specifying possible causes or effects of the system’s subsets in their current state. Each subset contributes to the intrinsic information of the whole to the extent that it is integrated, meaning irreducible under any partition. The total intrinsic information of a system thus corresponds to the compositional integrated information specified by the set of all of its mechanisms—not more and not less.

While we have restricted our analysis to a specific type of distributed dynamical system with finite state-space, the general argument—that a compositional perspective is necessary for understanding a system’s causal and information structure—should hold even in the case of an infinite state space and continuous time (see [46,47] for an initial approach to translate the principles of IIT to continuous dynamical systems). In that context, describing a system with a set of coupled differential equations, one per element (taking the place of the structural equations in Figure 1a), would correspond to a reductionist perspective, while a complete description of the system’s dynamics in global terms, e.g., via a Hamiltonian, would correspond to a holistic perspective (the phase portrait of such a system would then correspond to the state-transition diagram in Figure 1b). That the complexity of a system’s dynamics may increase through additional variables in a compositional manner is well-known in dynamical systems theory, where it is common practice to evaluate the nullclines and isoclines of a set of coupled differential equations, i.e., to evaluate the system’s dynamics while holding a subset of variables (or their slopes) fixed ([48]). In [47], Kalita et al. used a similar approach to assess the intrinsic information ($\varphi_{C/E}$) specified by the various subsets of a continuous dynamical system by example of a set of coupled Lotka-Volterra equations.

Finally, the compositional structure of a system is not just relevant intrinsically, but also matters in functional terms for systems that interact dynamically with an environment. Before discussing the role of composition for autonomy and agency below, we compare our approach to other approaches for information decomposition [39,40,41,42,49,50,51]. To conclude, we will review the compositionality of phenomenal consciousness and how it is addressed within IIT.

### 4.1. Composition vs. Decomposition of Information

Over the last decade, assessing the structure of multivariate information has become a focus within the field of complex system science. In a seminal paper, Williams and Beer [39] set out to decompose the Shannon information of a multivariate system into separate parts that reflect the unique, redundant, and synergistic information of its subsets about a target variable *S*. Several subsequent publications have aimed at improving upon this proposal of a partial information decomposition (PID) by refining the notion of redundancy and of synergy between variables [40,41,42,49,50,51].

Our approach differs from PID measures in several ways. First, we are interested in the *causal* information specified by the various subsets of a system *in a particular state*, not a decomposition of the mutual information between source and target variables in the joint distribution of an observed time series. $\varphi_{C}$ and $\varphi_{E}$ (Equation (15)) are state-dependent measures and evaluate whether a subset at time *t* specifies information about the system’s prior or next state, respectively. As shown in [52], PID can also be applied to decompose transfer entropy, a directional measure of information transfer from a variable $Y_{t}$ to another variable $X_{t+1}$, extended to the case of multiple sources. However, transfer entropy still relies on observational data, while a causal approach generally requires perturbational data [53] (although observational data is sufficient for causal inference in ER systems since they cycle through all their possible states). In this way, our approach is more closely related to proposed measures of causal information flow [44,54], but evaluated in a state-dependent manner, as the information specified by the subset in its current state about its causes and effects (see also [55]).

Second, from a causal perspective, two system subsets may both exert informationally redundant causal constraints, for example in cases of causal overdetermination [31,40]. While the notion of integration evaluated by $\varphi_{C}$ and $\varphi_{E}$ is related to the synergistic and unique parts in the PID, not all information that would be deemed redundant from an information-theoretical perspective is discounted in our approach. For instance, in the example system of Figure 1 and Figure 3, the (Shannon) information specified by $C_{t}$ and $X_{t}$ about the state of $MCX_{t+1}$ is redundant. Nevertheless, they both make a difference to the future state of MCX by raising the probability of $M_{t+1}=1$ in mechanistic, causal terms, and thus count toward the system’s intrinsic information $\sum \varphi_{C}+\sum \varphi_{E}$. Also, in our approach irreducibility is evaluated based on a partition of the subset (Equation (13)), which eliminates dependencies across the partition, rather than by comparing the subset to other subsets within the system (see also [56]).

Finally, as in [27], $\Phi_{\subseteq }$ evaluates the integrated information of the system as a whole as the amount of compositional intrinsic information lost through a system partition (see Equation (1) and Section 5.5). Consequently, $\Phi_{\subseteq }$ is not bound by the predictive information (3) of the system about its next state as the PID measures or also the geometrical integrated information measure proposed in [56], but rather by $\min\left(\sum \varphi_{C}\left(v_{t}\right),\sum \varphi_{E}\left(v_{t}\right)\right)$.

While the role of composition in accounting for the quality of phenomenal experience (see Section 4.3) had already been recognized in earlier publications [57], it was not incorporated in the quantitative measure $\phi_{2.0}$ [58]. Similarly, the geometric integrated information framework [56] permits the evaluation of partial causal influences and their hierarchical structure. However, the geometric integrated information of a system $\Phi_{G}$ still only takes the highest level into account. Moreover, $\Phi_{G}$ is an average, not a state-dependent measure. In Appendix B, we compare compositional and non-compositional measures of system-level integrated information. While non-compositional, state-averaged measures may serve as practical indicators for a system’s capacity for information integration, for a state-dependent evaluation the system’s causal composition cannot be neglected.

### 4.2. Agency and Autonomy

In the above analysis, we have treated each system as an isolated entity. Agents, however, are open systems that interact dynamically and informationally with their environment [59]. The global dynamics of an agent thus depend in some way on the state evolution of the environment. Conversely, “agency” implies that the system has some effect on the dynamical evolution of the environment. How should the environment be incorporated into an account that relies on the global dynamics of a system? And how can we identify the agent as an autonomous entity within the larger dynamical system?

**Figure 8:**
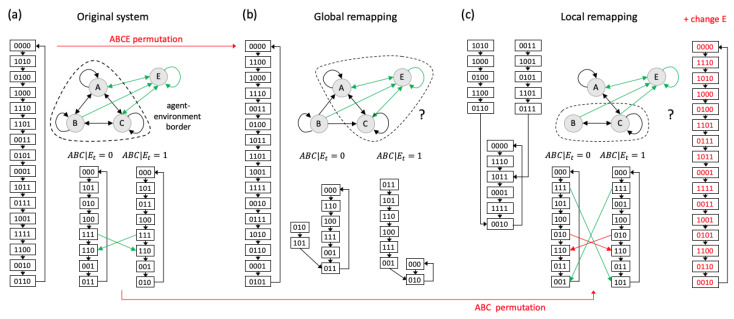
Dynamics of a joint agent–environment system. (**a**) The system ABC forms a hypothetical agent that interacts dynamically with its environment. ABCE forms a (4-node) ER system, as does ABC if *E* is taken as a fixed background condition. Element *E* changes its state whenever ABC=111. ABC is the subset with $\max(\Phi_{\subseteq })$ in all 16 states. We consider two cases of dynamical equivalence: (**b**) Permuting the states of ABCE in the global state-transition diagram will typically change the local dynamics of the agent subsystem ABC and the prior agent–environment division is lost. Note that *B* is connected to the rest of the system in a purely feedforward manner. Instead of ABC, now ACE forms the set of elements with $\max(\Phi_{\subseteq })$ in most states (11/16, discounting single elements). (**c**) A local remapping of the state-transition diagram of ABC will typically change the global dynamics, if the input-output function of the environment *E* remains unchanged. This changes the agent’s behavior with respect to its environment. In order to recover the global dynamics *E*’s mechanism needs to be adapted. Even in this case, however, the agent–environment division may not be maintained and BC is now the set of elements with $\max(\Phi_{\subseteq })$ in most (14/16) states.

In Figure 8, we consider a system ABCE in which the elements ABC stand for a hypothetical “agent” that dynamically interacts with its environment *E*. This example was constructed such that the joint system ABCE is an ER system, which cycles through all of its 16 possible states. In addition, ABC forms a 3-node ER system if the environment *E* is fixed to either of its possible states. We consider two cases of dynamical equivalence: in Figure 8b we permute the global dynamics of the joint agent–environment system ABCE, whereas in Figure 8c we permute the local dynamics of the agent ABC.

It is easy to show that, if we describe the joint agent–environment system in terms of its global dynamics, a permutation of the global states in the state-transition diagram will typically not maintain the dynamics of the agent-subsystem. Figure 8b shows an example of a different 4-node ER system with equivalent global dynamics that can be obtained by permuting the order in which ABCE in Figure 8a cycles through all its possible states. As the binary state labels have no meaning from a holistic perspective, such a permutation maintains the global system dynamics. In the permuted system (Figure 8b) however, the subsystem ABC, holding *E* fixed, is not reversible, but instead shows some convergence. This example demonstrates that the previous subdivision of ABCE into agent and environment is lost due to the global permutation, which changed the interactions between the system elements, including those between ABC and *E*. For example, node *B* in Figure 8b is now connected in a purely feedforward manner to the rest of the system and simply alternates its state between 0 and 1. Thus, from the perspective of AC and also *E*, *B* now merely forms a background condition, as ACE has no information, and thus no control over the next state of *B*.

Of course, this example also raises the question of why ABC was determined to be a separate entity from the environment *E* in the first place [23,33,35,60,61,62,63]. While the boundaries of an agent are typically taken as given, such a subdivision cannot be properly formulated using a reductionist or holistic account of the system’s dynamical or informational properties. The IIT formalism, on the other hand, provides the tools to identify subsets of elements with self-defined causal borders within larger systems as local maxima of integrated information [23,27,33,64] (see also [32,34] for alternative proposals). (In IIT as a theory of consciousness, a maximality condition is imposed by the “exclusion” postulate, which translates the fact that phenomenal experience is definite in its content into the requirement that also the underlying physical substrate must specify a definite set of mechanisms—one that forms a maximum of integrated information Φ). To illustrate, the dashed line in Figure 8a–c indicates the subset of elements with $\max(\Phi_{\subseteq })$ in the majority of states, respectively.

Instead of describing the joint agent–environment dynamics, it is also possible to treat the environment *E* as a fixed background condition. As demonstrated in Figure 8c, remapping the local state-transition diagram of ABC will typically change the global dynamics of ABCE if the input-output function of *E* and its connectivity to ABC remain unchanged. This means that replacing ABC with another system with an equivalent state-transition diagram effectively changes the “agent’s” input-output behavior. To recover the global dynamics, mechanisms within the environment would have to be changed in addition to the mechanisms within the system (see red state transition diagram in Figure 8c). Thus, replacing a subsystem with another that has an equivalent local state-transition diagram does have different functional consequences for the global system. From an evolutionary perspective, an agent has limited control over the causal structure of the environment. For this reason, some agent implementations will typically be advantageous over others even if, in theory, they are dynamically equivalent at the level of the agent subsystem.

Now consider the system in Figure 8c with the adapted environment (red state-transition diagram), which is dynamically equivalent to the system in Figure 8a both in terms of the global dynamics of ABCE, as well as the local dynamics of ABC. However, this joint agent–environment system is constituted of a set of elements that perform different functions and are connected in different ways, so that the dynamics of other subsystems within ABCE, such as AB, are not maintained. Thus, even under this permutation, the previous agent–environment division may disappear.

In general, to define an agent as an autonomous entity separate from the environment in objective terms requires a search across all possible system subsets. Given a quantitative measure of autonomy based on dynamical, informational, or causal criteria, agents can then be identified as subsystems that form local maxima of autonomy [27,32,33,34,65]. As long as not all subsystems have equivalent dynamics under a permutation of the states in the global state transition diagram, these maxima may correspond to different subsets of elements in the original and the permuted system. Thus, from the perspective of the agents within the system, such a global permutation is far from ontologically innocent.

Finally, when an agent interacts with its environment, we are often interested in *why* the agent performed a particular action. Due to recent advances in the field of artificial intelligence, there is a growing realization that the ability to predict what a system is going to do does not equal understanding how or why it behaves in a certain way, not even in hindsight (e.g., [66,67]). This is demonstrated particularly well by recent computational studies involving simulated artificial agents with minimal cognitive architectures [15,23,68,69], whose behavior can easily be predicted. Yet, understanding what caused the agent to perform a particular action typically requires extensive additional analysis and cannot be addressed in purely reductionist or holistic terms [31,69,70].

### 4.3. The Role of Composition in IIT as a Theory of Phenomenal Consciousness

Related to the notion of agency is the question when a system of interacting elements may form a conscious entity. A distinguishing feature of IIT as a theory of consciousness is that it starts from phenomenology itself, which is the one and only thing whose existence is directly evident to the experiencing entity [26]. Next, IIT aims to capture the essential properties common to all of our experiences, which form its set of “axioms” about phenomenology. IIT identifies “composition” as one of its five phenomenal axioms as every experience is structured, being composed of phenomenal distinctions and the relations among them. The other axioms are “intrinsicality”, “information”, “integration”, and “exclusion” [25,26,27]. According to IIT, for each essential property of experience, there must be a corresponding property of the physical substrate that is underlying the experience. These are specified in a set of “postulates”, which translate each axiom into a causal requirement about the physical substrate.

A useful example to illustrate the compositional nature of phenomenology is our experience of space, for example visual space, which is accompanied by a feeling of extendedness, being composed of a multitude of distinguishable “spots” of arbitrary sizes, which are related to each other through connection, inclusion, and union (see [71] and Haun and Tononi, submitted). From the intrinsic perspective of the system itself, spatial properties such as the particular region and location of a spot, its size, boundary, and distance from other spots, have to be established by the system’s own causal structure. A holistic description that only captures the information of the visual canvas as a whole cannot give an account of the immense number of phenomenally distinct spots within the scene and their relations. On the other hand, a reductionist description that captures only the individual spots cannot account for their composition into an extended canvas, with specific relations among them. From an extrinsic, information-theoretical perspective, the list of phenomenal distinctions about visual space that we experience directly contains a lot of redundant information. However, such a perspective takes space for granted and overlooks its qualitative properties.

IIT proposes that it is the compositional cause-effect structure specified by a physical substrate that corresponds one-to-one to its phenomenal experience [26,27] (see Haun and Tononi, submitted, for a demonstration of how the cause-effect structure of a simple grid-like substrate may account for the main phenomenal properties of spatial experience). Within IIT, understanding the causal composition of a system is thus necessary not only to capture the amount of integrated (intrinsic) information (Φ) specified by a system, but also to characterize the phenomenal content of its experience, namely its compositional structure.

## 5. Methods

As a simple type of (recurrent) neural network model, we consider the class of distributed dynamical systems constituted of a set of *n* interacting elements $S={\left\{S_{i}\right\}}_{i\in 1\ldots n}$ with finite state space $\Omega_{S}=\prod_{i}\Omega_{S_{i}}$ that evolve in discrete time according to the update functions of the individual system elements (Figure 1). *S* is assumed to be stationary, which means that its update function and connectivity do not change over time. We further assume that there is no instantaneous causation between system elements. Examples of this type of systems include cellular automata and Boolean networks. While we will restrict our analysis to systems constituted of binary elements, all quantities described below can equally be applied to systems with finite-valued elements.

As illustrated in Figure 1, the temporal evolution of such distributed dynamical systems can be specified within the framework of dynamical causal networks, as well as by means of their state transition probabilities.

### 5.1. Dynamical Causal Networks and State Transition Probabilities

Causal networks are a special type of Bayesian networks in which the edges represent causal dependencies as opposed to mere correlations. Specifically, a causal network G=(V,E) is a directed acyclic graph (DAG) with edges *E* that indicate causal connections between a set of random variables *V*, which also correspond to the nodes in the graph. Variables are equipped with an update function, or structural equation, which specifies the (probabilities of) a variable’s output state given the state of its inputs. The set of variables $pa\left(V_{i}\right)=\left\{V_{j}|e_{ji}\in E\right\}$ with an edge leading into $V_{i}\in V$ are called the “parents” of $V_{i}$.

As *G* is a Bayesian network, a probability function p(V=v) with $v\in \Omega_{V}$, is associated with the random variables *V*, such that:

$$p\left(v\right)=\prod_{i}p\left(v_{i}|pa\left(V_{i}\right)\right),\;v\in \Omega_{V}.$$

In a causal network, this conditional independence of individual variables holds even if the parents are actively set into their state, as opposed to being passively observed. (For simplicity, we assume that exogenous variables can be considered as fixed background conditions and thus do not have to be further taken into account in the causal analysis (see [31]).) This intervention can be indicated by the “do-operator” [53]:

$$p\left(v\right)=\prod_{i}p\left(v_{i}|do\left(pa\left(V_{i}\right)\right)\right),\;v\in \Omega_{V}.$$

In a dynamical causal network, all parents of the variables in slice $V_{t}$ are contained in the previous slice $V_{t-1}$ [31]. Together with the above, this requirement implies a transition probability function for *V*, such that:

(2) $$p\left(v_{t}|v_{t-1}\right)=\prod_{i}p(v_{i,t}|v_{t-1})=\prod_{i}p\left(v_{i,t}|do\left(v_{t-1}\right)\right)=p\left(v_{t}|do\left(v_{t-1}\right)\right),\;\forall \;\left(v_{t-1},v_{t}\right)\in \Omega .$$

Equation (2) fully captures the causal interactions between the set of variables *V*. If we interpret the dynamical causal network $G_{S}$ as a temporal unfolding of a discrete dynamical system *S* (Figure 1a), the probabilities in Equation (2) directly correspond to the system’s state transition probabilities. (While $p(v_{t}|v_{t-1})$ is generally not defined for $v_{t-1}$ with $p(v_{t-1})=0$, here we assume that the system can, at least in principle, be perturbed into all possible states. We can thus define $p\left(v_{t}|v_{t-1}\right)=p\left(v_{t}|do\left(v_{t-1}\right)\right)$ for all $v_{t-1}\in \Omega_{V_{t-1}}$, even if $p(v_{t-1})=0$.) Since *S* fulfills the Markov property and we assume stationarity, the system’s dynamics are completely specified by its one-time-step transition probability matrix (TPM) $M_{S}^{r,c}=p\left(v_{t}^{c}|v_{t-1}^{r}\right),\;\forall \left(v_{t-1},v_{t}\right)\in \Omega_{S}\times \Omega_{S}$, where $r,c\in \{1,\cdots ,|\Omega_{S}|\}$ are the row and column indices, respectively (Figure 1b). Conditional independence between individual system elements (Equation (2)) moreover permits us to represent the matrix in the state-by-node format for binary systems, as shown in Figure 1b on the right. The state-by-node table specifies the probability for each element to be in state ‘1’ given each possible prior system state.

Finally, note that, in a deterministic system, an element’s output is completely determined by the state of its input, and thus conditionally independent from the output of all other system elements. Therefore, all deterministic TPMs automatically comply with Equation (2). This is not generally the case for generic probabilistic TPMs, which may violate the “no instantaneous causation” requirement and thus do not comply with Equation (2), which also means that they cannot be expressed in state-by-node format.

For this reason, we will formulate all quantities defined below within the context of a dynamical causal network $G_{S}=\left(V,E\right)$ with $V={\left\{V_{t}\right\}}_{t\in \{0,\cdots ,k\}}$ and $V_{t}=S,\;\forall t\in \left\{0,\cdots ,k\right\}$ for maximal clarity, with reference to the system’s TPM when appropriate. For clarity, we will denote probability distributions as functions of variables, e.g., p(X), and individual probabilities as functions of states, e.g., p(x). We use *S* to denote the system in general, when we refer to the set of interacting elements, but write $V_{t}$ to denote the set of all system elements at a particular point in time *t*.

### 5.2. Predictive and Effective Information

The mutual information I(X;Y) between two sets of random variables *X* and *Y* can be expressed as a difference in entropy:

I(X;Y)=H(Y)−H(Y|X)=H(X)−H(X|Y),

where $H\left(X\right)=\sum_{x\in \Omega_{X}}p\left(x\right)\log_{2}p\left(x\right)$ and $H\left(X|Y\right)=\sum_{\left(x,y\right)\in \Omega_{X\times Y}}p\left(x,y\right)\log_{2}p\left(x,y\right)/p\left(y\right)$, with $0*log_{2}\left(0\right):=0$. I(X;Y) thus captures the expected reduction of uncertainty about the state of *Y* given the state of *X* and vice versa. Mutual information is symmetric with I(X;Y)=I(Y;X) and non-negative I(X,Y)>0. In general, I(X;Y) is computed from a joint probability distribution p(X,Y) of interest, which is typically sampled from observed time series data. The mutual information between two consecutive system states of a time series has been termed *predictive information* [38]. Within the dynamical causal network $G_{S}$, the predictive information between $V_{t}$ and $V_{t-1}$ can also be expressed in terms of the transition probabilities specified in Equation (2):

(3) $$I\left(V_{t-1};V_{t}\right)=H\left(V_{t}\right)-H\left(V_{t}|V_{t-1}\right)=\sum_{v_{t-1}\in \Omega_{S}}p\left(v_{t-1}\right)D_{KL}(p\left(V_{t}|v_{t-1}\right)||p\left(V_{t}\right))$$

using the equivalent formulation of the mutual information as the expected *Kullback–Leibler divergence* $D_{KL}$, also called *relative entropy*, between the conditional probability distribution $p(V_{t}|v_{t-1})$ and the marginal distribution $p\left(V_{t}\right)=\sum_{v_{t-1}\in \Omega_{S}}p\left(v_{t-1}\right)p\left(V_{t}\right|p\left(v_{t-1}\right)$, where

(4) $$D_{KL}\left(p\left(Y|x\right)||p\left(Y\right)\right)=\sum_{y\in \Omega_{Y}}p\left(y|x\right)\log_{2}\frac{p(y|x)}{p(y)}.$$

Note that $D_{KL}\left(p\left(Y|x\right)||p\left(Y\right)\right)$ depends on the state of *X*. $G_{S}$ specifies causal dependencies between $V_{t-1}$ and $V_{t}$, with fixed transition probabilities $p(v_{t}|v_{t-1})$. In addition, the predictive information $I(V_{t-1};V_{t})$ for a particular $G_{S}$ depends on the choice of $p(V_{t-1})$. A typical choice is the stationary observed distribution of system states given a particular initial condition. In that case, $I(V_{t-1};V_{t})$ measures the predictability of the next system state $v_{t}$ following an observation of state $v_{t-1}$ in a particular dynamical regime of system *S*. Another useful choice in the context of dynamical causal networks, is to impose a uniform, or maximum entropy, distribution with $p\left(v_{t-1}\right)={\left|\Omega_{S}\right|}^{-1}$, $\forall v_{t-1}\in \Omega_{S}$. In this way, one obtains a measure of the causal constraints imposed by $G_{S}$, independent of any biases in the initial distribution of $p(V_{t-1})$. This measure has been termed the *effective information* EI(S) of a discrete dynamical system *S* [36,37].

(5) $$EI\left(S\right)=|\Omega_{S}{|}^{-1}\sum_{v_{t-1}\in \Omega_{S}}D_{KL}(p\left(V_{t}|v_{t-1}\right)||p\left(V_{t}\right))$$

can be conveniently represented in terms of the system’s TPM, as it corresponds to the average $D_{KL}$ between the distribution specified by each row $M_{S}^{r}=p\left(V_{t}|v_{t-1}^{r}\right)$ in $M_{S}$ and $p(V_{t})$, which corresponds to the distribution that results from averaging (“causally marginalizing” (see below)) across all rows in $M_{S}$ (see Figure 4). By contrast to the predictive information, EI(S) has a causal character, as imposing a maximum entropy distribution on $p(V_{t})$ corresponds to perturbing the system in all possible ways as $p\left(v_{t}|v_{t-1}\right)=p\left(v_{t}|do\left(v_{t-1}\right)\right)$ (Equation (2)) [37]. As a consequence, any measured constraints on $p(V_{t})$ are *intrinsic*, i.e., due to the system’s mechanisms and nothing else. In the following, we will expand on the notion of *intrinsic information* by defining the information that a system specifies onto itself in a state-dependent and compositional manner.

### 5.3. Cause and Effect Repertoires

By being in state $v_{t}$, the system *S* constrains its potential next states according to its state transition probabilities $p\left(v_{t+1}|v_{t}\right)=p\left(v_{t+1}|do\left(v_{t}\right)\right)$ (Equation (2), assuming stationarity). We can define the *effect repertoire* of $v_{t}$ as:

(6) $$\pi \left(V_{t+1}|v_{t}\right)=p\left(V_{t+1}|do\left(v_{t}\right)\right)=p\left(V_{t+1}|v_{t}\right).$$

Likewise, for any state $v_{t}$ with $p(v_{t})>0$, the system also constrains its potential prior states and we can infer the reverse conditional probabilities from Equation (2) by using Bayes’ theorem: $p\left(v_{t-1}|v_{t}\right)=p\left(v_{t}|v_{t-1}\right)*p\left(v_{t-1}\right)/p\left(v_{t}\right)$. Here, $p(v_{t-1})$ is meant to represent the prior probability of $V_{t-1}=v_{t-1}$ in the absence of any constraints due to the system’s mechanisms or present state, and not the probability that $v_{t-1}$ occurs under any observed or imposed state distribution. Given the system’s state transition probabilities (Equation (2)) and the present state $v_{t}$ of the system, the intrinsic causal constraints specified by the system itself should not depend on any further external factors, or prior system states. For this reason, the appropriate choice for $p(V_{t-1})$ is, again, to impose a uniform distribution with $p\left(v_{t-1}\right)={\left|\Omega_{S}\right|}^{-1}$, $\forall v_{t-1}\in \Omega_{S}$. This avoids any biases or assumptions about $p(V_{t-1})$ that are not intrinsic, i.e., unavailable to the system itself [27,31,37]. Together with Equation (2), it follows that $p\left(v_{t}\right)={\left|\Omega_{S}\right|}^{-1}\sum_{v_{t-1}\in \Omega_{S}}p\left(v_{t}|do\left(v_{t-1}\right)\right)$, $\forall v_{t}\in \Omega_{S}$. Imposing a uniform distribution for $p(V_{t-1})$, moreover, corresponds to the notion of *causal marginalization* [31], which means averaging across all possible states of those variables that are not conditioned to any particular state. Taken the above into account, we define the *cause repertoire* of $v_{t}$ as:

(7) $$\pi \left(V_{t-1}|v_{t}\right)=\frac{p(v_{t}|do\left(V_{t-1}\right))}{\sum_{v_{t-1}\in \Omega_{S}}p\left(v_{t}|do\left(v_{t-1}\right)\right)}=\frac{p(v_{t}|V_{t-1})}{\sum_{v_{t-1}\in \Omega_{S}}p\left(v_{t}|v_{t-1}\right).}$$

Following [31], we denote cause and effect repertoires by π, as their general definition (below) is not equivalent to a simple conditional probability distribution in all cases. Moreover, conditional probability distributions are typically derived from a freely chosen joint distribution. By contrast, causal marginalization corresponds to imposing a uniform distribution on $p(V_{t-1})$ in the definition of the cause repertoire (or, respectively, $p(V_{t})$ for the effect repertoire).

Not only the system as a whole, but also its parts, that is, all subsets X⊆S, may specify information about the system’s potential prior and next states by being in their particular present state $x_{t}$ (Figure 3). As described in detail in [25,27,31], the cause and effect repertoire of a subset X⊆S in state $x_{t}\subseteq v_{t}$ can be obtained from the system’s transition probabilities (Equation (2)) by conditioning on $x_{t}$ and causally marginalizing the variables $W_{t}=V_{t}\backslash X$. The goal is to remove any contributions of $W_{t}$ to the repertoire by averaging over all possible states of $W_{t}$. However, common inputs from variables in $W_{t}$ may still introduce biases in the state distribution of $V_{t+1}$. To discount such correlations, the effect repertoire of $x_{t}$ over $V_{t+1}$ is computed as the product of the effect repertoires of $x_{t}$ over each individual variable $V_{i,t+1}\in V_{t+1}$ [27,31,54]. More generally, within $G_{S}$, the effect repertoire of X⊆S in its present state $x_{t}\subseteq v_{t}$ on a subset $Z_{t+1}\in V_{t+1}$ is defined as:

(8) $$\pi \left(Z_{t+1}|x_{t}\right)=\prod_{i}\pi \left(Z_{i,t+1}|x_{t}\right)=\prod_{i}\frac{1}{|\Omega_{W}|}\sum_{w\in \Omega_{W}}p\left(Z_{i,t+1}|do\left(x_{t},W_{t}=w_{t}\right)\right).$$

In this way, all variables in $Z_{t+1}$ are conditioned on $x_{t}$, but receive independent “random” inputs from $W_{t}$.

Likewise, the cause repertoire of a system subset X⊆S in its present state $x_{t}\subseteq v_{t}$ on a subset $Z_{t-1}\in V_{t-1}$ is defined as:

(9) $$\pi \left(Z_{t-1}|x_{t}\right)=\frac{\prod_{i}\pi \left(Z_{t-1}|x_{i,t}\right)}{\sum_{z\in \Omega_{Z_{t-1}}}\prod_{i}\pi \left(Z_{t-1}=z|x_{i,t}\right)}$$

where the product now is over the individual variables $X_{i}\in X$ with:

(10) $$\pi \left(Z_{t-1}|x_{i,t}\right)=\sum_{y\in \Omega_{Y_{t-1}}}\frac{p\left(x_{i,t}|do\left(Z_{t-1},Y_{t-1}=y\right)\right)}{\sum_{v_{t-1}\in \Omega_{S}}p\left(x_{i,t}|do\left(v_{t-1}\right)\right)}.$$

Here, the outer sum corresponds to the causal marginalization of $Y_{t-1}=V_{t-1}\backslash Z_{t-1}$, while the term inside is equivalent to Equation (7) and follows from applying Bayes’ theorem to $\pi (Z_{t-1}|x_{i,t})$. By computing $\pi (Z_{t-1}|x_{i,t})$ as the product over individual $X_{i}$ in Equation (10), we discount potential biases due to common inputs from $Y_{t-1}=V_{t-1}\backslash Z_{t-1}$ to variables in *X*.

Note that Equation (8) reduces to Equation (6) in the case that $X=Z_{t+1}=S$, and Equation (9) reduces to Equation (7) in the case that $X=Z_{t-1}=S$ because of the conditional independence specified in Equation (2). In general, however, $\pi \left(Z_{t+1}|x_{t}\right)\neq p\left(Z_{t+1}|x_{t}\right)$ and also $\pi \left(Z_{t-1}|x_{t}\right)\neq p\left(Z_{t-1}|x_{t}\right)$. For the purpose of comparison, we can also define *unconstrained* cause and effect repertoires $\pi (Z_{t-1}))$ and $\pi (Z_{t+1}))$ which can be derived from Equations (9) and (8) by using the convention that π(⌀)=1 [31], specifically:

(11) $$\pi \left(Z_{t-1}\right)={\left|\Omega_{Z_{t-1}}\right|}^{-1}$$

and

(12) $$\pi \left(Z_{t+1}\right)=\prod_{i}\pi \left(Z_{i,t+1}\right)=\prod_{i}{\left|\Omega_{S}\right|}^{-1}\sum_{v_{t-1}\in \Omega_{S}}p\left(Z_{i,t+1}|do\left(v_{t-1}\right)\right).$$

Given the above definitions, we can quantify the amount of information specified by a subset $X_{t}=x_{t}$ of the system about the possible prior or next state of other subsets $Z_{t\pm 1}$ as the difference between the respective cause or effect repertoire and the corresponding unconstrained cause or effect repertoire. The subset $Z_{t\pm 1}$ over which the causal constraints of $x_{t}$ are evaluated is called the cause or effect “purview”, respectively. Within the IIT formalism, an appropriate difference measure (in line with IIT’s axioms) should be used for this purpose [27] (see also Barbosa et al., in prep, for a novel *intrinsic* distance measure derived from first principles to comply with IIT’s axioms). Here, however, we want to remain as close as possible to standard measures of information theory and thus evaluate the difference between the repertoires using the Kullback–Leibler divergence $D_{KL}$ (Equation (4)).

### 5.4. Subset Integration

**Figure 9:**
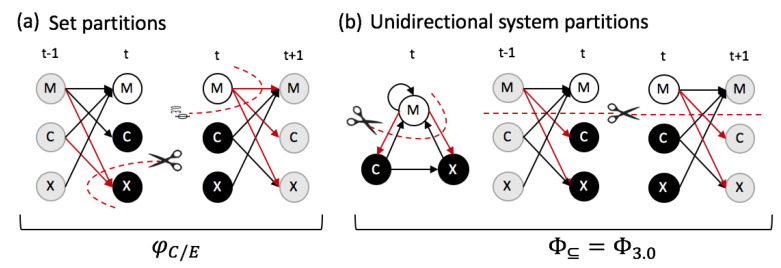
Permissible partitions. (**a**) To assess the integrated intrinsic information $\varphi_{C/E}\left(x_{t}\right)$ specified by a subset of system elements X⊆S at *t* about the prior or next states of the system, $x_{t}$ has to be partitioned into at least two parts, here, e.g., $\left\{\left({\left(MCZ\right)}_{t-1}|{\left(MC\right)}_{t}\right)\times \left(⌀|X_{t}\right)\right\}$ and $\left\{\left(M_{t+1}|M_{t}\right)\times \left({\left(CX\right)}_{t+1}|{\left(CX\right)}_{t}\right)\right\}$. (**b**) Unidirectional system partitions as defined in [27]. The connections from one part of the system to another (but not vice versa) are partitioned.

As exemplified in Figure 3, the various subsets of a system may specify qualitatively different information about the possible prior and next states of other subsets within the system. However, a subset only contributes to the intrinsic information of the system as a whole to the extent that it forms an irreducible (integrated) mechanism within the system. This means that a partition of the subset must affect its cause or effect repertoire and thus the amount of information it specifies about the system’s prior or next states. Otherwise the subset is reducible to its parts. Following [31], a partition ψ of a subset X⊆S in its current state $x_{t}$ (and the nodes it constrains $Z_{t\pm 1}$) into *m* parts is defined as:

(13) $$\psi \left(Z_{t\pm 1},x_{t}\right)=\left\{\left(Z_{1,t\pm 1},x_{1,t}\right),\left(Z_{2,t\pm 1},x_{2,t}\right),\ldots ,\left(Z_{m,t\pm 1},x_{m,t}\right)\right\},$$

such that ${\left\{x_{i,t}\right\}}_{i=1}^{m}$ is a partition of $x_{t}$ and $Z_{j,t\pm 1}\subseteq Z_{t\pm 1}$ with $Z_{j,t\pm 1}\cap Z_{k,t\pm 1}=⌀,\;j\neq k$. Note that this includes the possibility that any $Z_{j,t\pm 1}=⌀$, which may leave a set of nodes $Z_{t\pm 1}\backslash {⋃}_{j=1}^{m}Z_{j,t\pm 1}$ completely unconstrained (see Figure 9a for examples and [31] for further details). Defined as in Equation (13), the partition necessarily eliminates the possibility of joint constraints from $x_{t}$ onto $Z_{t\pm 1}$.

Next, the partition ψ is applied to the cause or effect repertoire of $x_{t}$. The partitioned repertoire is the product of the cause/effect repertoires of the *m* parts, multiplied by the unconstrained effect repertoire (Equations (11) and (12)) of the remaining set of nodes $Z_{t\pm 1}\backslash {⋃}_{j=1}^{m}Z_{j,t\pm 1}$, as these nodes are no longer constrained by any part of $x_{t}$ under the partition:

(14) $$\pi^{\psi }\left(Z_{t\pm 1}|x_{t}\right)=\prod_{j=1}^{m}\pi \left(Z_{j,t\pm 1}|x_{j,t}\right)\times \pi \left(Z_{t\pm 1}\backslash \overset{m}{\underset{j=1}{⋃}}Z_{j,t\pm 1}\right).$$

The irreducible cause or effect information $\varphi_{C/E}\left(x_{t}\right)$ of a subset X⊆S in its current state $x_{t}$ for a particular partition ψ can then be obtained by comparing the intact cause or effect repertoire to the partitioned cause or effect repertoire. Of all partitions, the one that makes the least difference to the cause/effect repertoire (termed “MIP” for minimum information partition) determines the value of $\varphi_{C/E}$ for a given $x_{t}$ over purview $Z_{t\pm 1}$.

Within the full IIT framework [25,27], the final value of $\varphi_{C/E}\left(x_{t}\right)$ depends on multiple additional factors, including the preferred difference measures [27] (see also Barbosa et al., in preparation), as well as a search across all possible purviews, the sets of elements $Z_{t\pm 1}\subseteq V_{t\pm 1}$, for the one that yields the highest $\varphi_{C/E}\left(x_{t}\right)$.

For our present purposes, however, the goal is to simplify the analysis as much as possible, in line with standard information theoretical considerations. For this reason, we again chose $D_{KL}$ (Equation (4)) as our difference measure. In combination with the particular set of permissible partitions (Equation (13)), the choice of $D_{KL}$ has the additional advantage that $\varphi_{C/E}\left(x_{t}\right)$ takes its maximal value for $Z_{t\pm 1}=V_{t\pm 1}$. This is because $D_{KL}$ is additive and any elements in $V_{t\pm 1}$ that are not constrained by $x_{t}$ simply add nothing to $\varphi_{C/E}\left(x_{t}\right)$.

Taken together, we can thus define the amount of integrated intrinsic information specified by a set of elements X⊆S in its current state $x_{t}$ as:

(15) $$\varphi_{C/E}\left(x_{t}\right)=\varphi_{C/E}\left(x_{t},\mathrm{MIP}\right)=D_{KL}\left(\pi \left(V_{t\pm 1}|x_{t}\right)\left|\right|\pi^{\mathrm{MIP}}\left(V_{t\pm 1}|x_{t}\right)\right).$$

For single variable subsets, where $x_{t}$ cannot be partitioned into m≥2 parts, $\varphi_{C/E}\left(x_{t}\right)$ simply amounts to the total amount of intrinsic information, as compared to the unconstrained cause or effect repertoire $\pi (V_{t\pm 1})$.

Using this simplified procedure, the cause and effect purviews ($Z_{t\pm 1}$) of $x_{t}$ then correspond to the full set of elements that are constrained by $x_{t}$, excluding only those system elements over which $x_{t}$ does not specify any information. In the full analysis, which does not use $D_{KL}$ as the difference measure of choice, the purviews can constitute subsets of this set.

### 5.5. System Integration

The intrinsic information of the system *S* as a whole in its current state $v_{t}$ is composed of the intrinsic information of its various integrated subsets. The exhaustive IIT formalism requires each subset $x_{t}$ to specify both $\varphi_{C}\left(x_{t}\right)>0$ and $\varphi_{E}\left(x_{t}\right)>0$, and only counts the minimum of the two values as the integrated information of the subset [25,27] within the system. Here, we simply sum all the integrated cause and effect information specified by each subset to obtain the total amount of intrinsic information available to the system: $\sum_{x_{t}\subseteq v_{t}}\varphi_{C}\left(x_{t}\right)+\sum_{x_{t}\subseteq v_{t}}\varphi_{E}\left(x_{t}\right)$, or short $\sum \varphi_{C}+\sum \varphi_{E}$.

A system exists as an integrated whole in its current state only if all its parts specify integrated information about the prior and next states of the rest of the system. This is evaluated by partitioning the connections from one part of the system X⊆S to the rest (Figure 9b): Ψ=X↛S\X, as defined in [25,27]. For each subset $x_{t}\subseteq v_{t}$ with $\varphi_{C/E}\left(x_{t}\right)>0$, the integrated information of the subset is reevaluated in the partitioned system:

(16) $$\varphi_{C/E}^{\Psi }\left(x_{t}\right)=D_{KL}\left(\pi^{\Psi }\left(V_{t\pm 1}|x_{t}\right)\left|\right|\pi^{\Psi +\mathrm{MIP}}\left(V_{t\pm 1}|x_{t}\right)\right).$$

The superscript “Ψ+MIP” signifies that on top of the system partition Ψ, the repertoire is partitioned according to the subset partition ψ (Equation (13)) that makes the least difference to $\pi^{\Psi }\left(V_{t\pm 1}|x_{t}\right)$. Next, the difference $\Delta \varphi_{C/E}\left(x_{t}\right)=\varphi_{C/E}\left(x_{t}\right)-\varphi_{C/E}^{\Psi }\left(x_{t}\right)$ is summed up separately for $\varphi_{C}$ and $\varphi_{E}$ across all possible subsets $x_{t}\subseteq v_{t}$ with $\varphi_{C/E}\left(x_{t}\right)>0$, which we denote as $\sum \Delta \varphi_{C}\left(v_{t}\right)$ and $\sum \Delta \varphi_{E}\left(v_{t}\right)$. Having defined these quantities, we obtain the definition of $\Phi_{\subseteq }$ (Equation (1)), a simplified compositional version of the canonical Φ [27], by taking the minimum between $\sum \Delta \varphi_{C}\left(v_{t}\right)$ and $\sum \Delta \varphi_{E}\left(v_{t}\right)$, and also across all possible partitions Ψ. $\Phi_{\subseteq }$ thus measures the minimal amount of compositional intrinsic information about the possible prior or next state of the system that is lost under any partition Ψ.

To summarize, compared to the canonical IIT formalism as described in [27], here we simplify the Φ computation in the following ways:

- We use the KLD to quantify differences between probability distributions in order to facilitate the comparison to standard information-theoretical approaches.
- The set of partitions evaluated to determine φ (Equation(13)) corresponds to the definition in [31], which provides an update compared to [27].
- For simplicity and in line with information-theoretical considerations, $\sum \varphi_{C}$ and $\sum \varphi_{E}$ are considered independently instead of only counting $\varphi =\min(\varphi_{C},\varphi_{E})$ for each subset.
- $\Phi_{\subseteq }$ simply evaluates the minimal difference in $\sum \varphi_{C}$ or $\sum \varphi_{E}$ under all possible system partitions instead of a more complex difference measure between the intact and partitioned system, such as the extended earth-mover’s distance used in [27].

### 5.6. Data Sets

To highlight the role of composition in the simplest possible terms, we focus on dynamical causal networks constituted of three binary elements. Note, however, that all measures specified above can in principle be applied to any causal network comprised of binary or multi-valued variables as long as it complies with Equation (2) [31].

To illustrate the expected range of intrinsic information and system-level integration specified by a random sample of systems, we evaluated two sets of 10,000 random matrices with either probabilistic or deterministic transition probabilities (see Appendix A). We created a random sample of 10,000 deterministic TPMs, as in Figure 1b, by assigning each input state at t−1 a randomly drawn output state at *t*. The random sample of 10,000 random probabilistic systems was generated by filling each entry in the state-by-node TPM (Figure 1b, right) with a random number between 0 and 1 drawn from a uniform distribution.

In order to disentangle the notion of composition as much as possible from other informational or dynamical system properties we further restrict ourselves to the set of reversible systems, and, in particular, the subset of ergodic reversible systems (Figure 4). In the present context, *reversible* is defined as follows:

**Definition** **1.** *A discrete dynamical system S with state space $\Omega_{S}$ and the associated dynamical causal network $G_{S}$ is reversible if $\forall s\in \Omega_{S},\;\exists z\in \Omega_{S}$:*

*1.*  *$p(v_{t-1}=z|v_{t}=s)=1$, and*
*2.*  *$p(v_{t}=s|v_{t-1}=z)=1$.*

Note that condition 1 is fulfilled by all deterministic systems. With respect to the transition probability matrix, condition 2 means that there is only a single ‘1.’ in each column. All such reversible systems specify the maximal value of effective information, EI(S)=n bit [37], which directly follows from conditions 1 and 2. In words, in a reversible system, every state is reachable and completely specifies the prior and next state of the system. In dynamical terms, however, reversible systems can still demonstrate a number of qualitatively different attractor landscapes with different numbers of fixed points and periodic cycles, leading to distinct observed, or stationary probability distributions depending on the initial state of the system (Figure 4a). For this reason, we specifically consider the subset of ergodic reversible (ER) systems, which transition through all possible system states over time:

**Definition** **2.** *A reversible system S with state space $\Omega_{S}$ and the associated dynamical causal network $G_{S}$ is ergodic if $\forall s,z\in \Omega_{S}$ with s≠z, $\exists d\in \{1,\cdots ,|\Omega_{S}|-1\}$: $p(v_{t+d}=z|v_{t}=s)=1$.*

The observed, stationary probability distribution p(S) of an ER system, approximates a uniform distribution over time. This means that for all ER systems the predictive information approximates the system’s effective information: $I\left(V_{t-1};V_{t}\right)≃EI\left(S\right)=n$ bit [37] for all initial conditions. In addition, all conditional entropies within p(S) (the joint distribution at one particular point in time) equal maximum entropy. Note, however, that the set of conditional entropies specified in Equation (2), which define the dynamical causal network of *S*, still differ for all unique ER systems. From a holistic perspective, however, all ER systems are dynamically identical, as they each follow a single periodic cycle through $\Omega_{S}$. In total, there are 40,320 distinct binary reversible systems of three interacting elements. Of these, 5040 are ergodic.

### 5.7. Software and Data Analysis

All quantities evaluated in this article were computed using custom-made python scripts (available upon request) based on PyPhi, the IIT python software package [72]. The particular version of PyPhi used can be found here: https://github.com/grahamfindlay/pyphi.git (commit: b79b7fa on branch ‘iit-4.0’, date: 03/29/2019). To compute $\sum \varphi_{C}+\sum \varphi_{E}$ and $\Phi_{\subseteq }$ the following non-standard settings were used in the pyphi.config file: ‘MEASURE’ = ‘KLD’, ‘PARTITION_TYPE’: ‘ALL’, ‘PICK_SMALLEST_PURVIEW’: True, and ‘USE_SMALL_PHI_DIFFERENCE_FOR_CES_DISTANCE’: ‘True’. Custom-made Matlab scripts were used for subsequent data analysis. Spearman rank correlation coefficients were used to evaluate correlations between measured quantities as the relation between the evaluated variable pairs is not necessarily linear. All obtained correlation values were highly significant ($p\ll 10^{-6}$) given the large sample sizes.

## Figures and Tables

**Table 1:** Irreducible information (in bits) specified by the subsets of $MCX_{t}=\left(0,1,1\right)$.

Subset	$M_{t}=0$	$C_{t}=1$	$X_{t}=1$	${\mathrm{MC}}_{t}=\left(0,1\right)$	${\mathrm{MX}}_{t}=\left(0,1\right)$	${\mathrm{CX}}_{t}=\left(1,1\right)$	${\mathrm{MCX}}_{t}=\left(0,1,1\right)$	$\sum \varphi_{C/E}$
$\varphi_{C}$	1.0	1.0	1.0	1.0	0.415	1.0	0.0	5.41
$\varphi_{E}$	1.189	0.189	0.189	1.0	0.0	0.415	0.415	3.40

**Table 2:** Irreducible information (in bits) specified by the subsets of the example systems in Figure 6 in state (0,1,1). Which sets specify irreducible information and how much they specify is state-dependent. Values of φ=0.0 bits are omitted for ease of comparison.

Subset $x_{t}$	$\varphi_{C}$				$\varphi_{E}$			
	(a)	(b)	(c)	(d)	(a)	(b)	(c)	(d)
$A_{t}=0$	1.0	1.0	1.0	1.0	1.0	0.189	1.0	0.566
$B_{t}=1$	1.0	1.0	1.0	1.0	1.0	0.189	0.378	0.566
$C_{t}=1$	1.0	1.0	1.0	1.0	0.189	1.0	0.378	0.566
$AB_{t}=\left(0,1\right)$	0.415		0.415	1.0		1.415	0.415	0.415
$AC_{t}=\left(0,1\right)$		1.0	0.415	0.83			0.415	0.415
$BC_{t}=\left(1,1\right)$	0.5	0.415	0.915	0.83		0.415	0.415	0.415
$ABC_{t}=\left(0,1,1\right)$			0.415	1.0	1.0		0.415	0.83
$\sum \varphi_{C/E}$	3.92	4.42	5.16	6.66	3.19	3.21	3.42	3.77

**Table 3:** Comparing the predictions $({\mathrm{argmax}}_{z_{t+1}}\left(p\left(z_{t+1}|x_{t}\right)\right)$ of irreducible subsets within the example systems in Figure 6a,d in state (0,1,1). The actual state at t+1 is (1,1,1) for the system in Figure 6a and (1,0,1) for Figure 6d.

Subset $x_{t}$	(a)		(d)	
	$z_{t+1}$	$p(z_{t+1}|x_{t})$	$z_{t+1}$	$p(z_{t+1}|x_{t})$
$A_{t}=0$	$A_{t+1}=1$	(p=1)	$ABC_{t+1}=\left(1,0,0\right)$	(p=0.42)
$B_{t}=1$	$C_{t+1}=1$	(p=1)	$ABC_{t+1}=\left(1,1,1\right)$	(p=0.42)
$C_{t}=1$	$B_{t+1}=0$	(p=0.75)	$ABC_{t+1}=\left(0,0,1\right)$	(p=0.42)
$AB_{t}=\left(0,1\right)$			$A_{t+1}=1$	(p=1)
$AC_{t}=\left(0,1\right)$			$B_{t+1}=0$	(p=1)
$BC_{t}=\left(1,1\right)$			$C_{t+1}=1$	(p=1)
$ABC_{t}=\left(0,1,1\right)$	$ABC_{t+1}=\left(1,1,1\right)$	(p=1)	$ABC_{t+1}=\left(1,0,1\right)$	(p=1)

**Figure A1:**
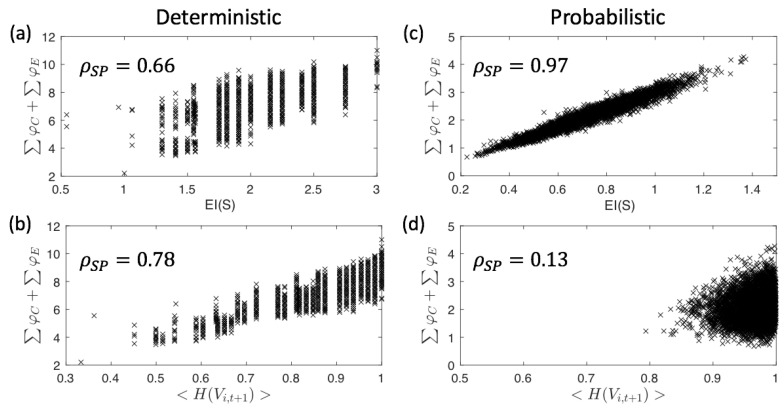
Intrinsic cause and effect information in a random sample of 10,000 binary 3-node systems. (**a**,**b**) Deterministic systems. (**c**,**d**) Probabilistic systems. (**a**,**c**) Correlation between EI(S) and the total amount of φ: $\sum \varphi_{C}+\sum \varphi_{E}$, again averaged across all possible system states. (**b**,**d**) Correlation between the average entropy $\langle H\left(V_{i,t+1}\right)\rangle$ of the individual system elements at t+1 and the total amount of φ. $\rho_{SP}$ is the Spearman rank correlation coefficient. Note that $\rho_{SP}\left(EI\right)$ is high for both deterministic and probabilistic systems, as EI(S) is a causal measure. By contrast, $\rho_{SP}\left(\langle H\left(V_{i,t+1}\right)\rangle \right)$ is high only for deterministic, not for probabilistic systems. This is because in probabilistic systems a large part of $\langle H\left(V_{i,t+1}\right)\rangle$ is explained by noise, while in deterministic system $\langle H\left(V_{i,t+1}\right)\rangle$ is due to the system’s mechanisms only.

**Figure A2:**
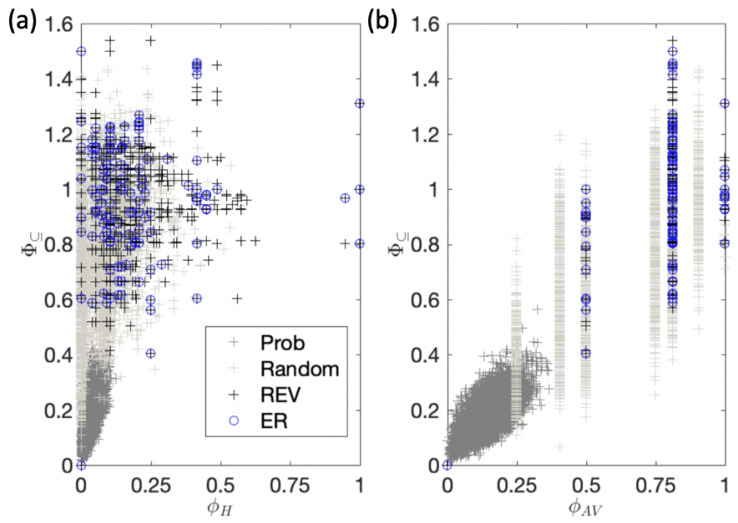
Non-compositional integrated information. (**a**) $\Phi_{\subseteq }$ is plotted against $\phi_{H}$. The measures are weakly correlated with $\rho_{SP}\left(\Phi_{\subseteq },\phi_{H}\right)=0.24$ for reversible systems, $\rho_{SP}\left(\Phi_{\subseteq },\phi_{H}\right)=0.47$ for the random deterministic sample, and more strongly correlated in the random probabilistic sample $\rho_{SP}\left(\Phi_{\subseteq },\phi_{H}\right)=0.58$. (**b**) $\phi_{AR}$ is plotted against $\Phi_{\subseteq }$. The correlation between $\Phi_{\subseteq }$ and $\phi_{AR}$ is stronger than for $\phi_{H}$, with $\rho_{SP}\left(\Phi_{\subseteq },\phi_{AV}\right)=0.48$ for reversible systems, $\rho_{SP}\left(\Phi_{\subseteq },\phi_{AR}\right)=0.75$ for the random deterministic sample, and $\rho_{SP}\left(\Phi_{\subseteq },\phi_{AR}\right)=0.69$ for the random probabilistic sample. Note that $\phi_{AV}$ only takes on a few discrete values in the evaluated deterministic systems. Moreover, $\phi_{AR}=0$ whenever $\Phi_{\subseteq }=0$ and not otherwise.

## Appendix A. Correlation between *EI*(*S*), 〈*H*(*V _i,t+1_*)〉, and ∑*_φC_* +∑*_φE_*

Reversible systems all specify a maximum amount of EI(S) (Equation (5)) and entropy upon perturbation. In deterministic systems, $EI\left(S\right)=H\left(V_{t+1}\right)$ (the entropy of the system at t+1 after imposing a uniform distribution of system states at time *t*) [36,37], because in deterministic systems $H(V_{t}|V_{t-1})=0$ in Equation (5). Similarly, we can define the average entropy $\langle H\left(V_{i,t+1}\right)\rangle$ of the individual system elements at t+1, again assuming a uniform distribution at *t*. EI(S) and $\langle H\left(V_{i,t+1}\right)\rangle$ are related to the differentiation measures $D_{1}$ and $D_{2}$ described in [73]. In line with [73], we found that both measures correlate with $\sum \varphi_{C/E}$ with $\rho_{SP}\left(EI\right)=0.654/0.4697$ and $\rho_{SP}\left(\langle H\left(V_{i,t+1}\right)\rangle \right)\;=\;0.769/0.579$, respectively. The overall strongest correlation was observed with the total amount of irreducible information $\sum \varphi_{C}+\sum \varphi_{E}$, displayed in Figure A1b,c, which is slightly higher than for $\sum \varphi_{C}$ alone.

By evaluating the informational composition of a system, we assess how the various parts of the system constrain its prior and next states. Using $D_{KL}$ as a distance measure, the irreducible information φ essentially quantifies how much of the system’s entropy is reduced by the various parts of the system in a compositional manner. In deterministic systems, the entropy of the system and its elements at t+1 (given a uniform distribution at *t*), is entirely due to the system’s causal mechanisms. Taken together, this explains the strong correlation between $\sum \varphi_{C}+\sum \varphi_{E}$ and the entropy measures.

Correlation of $\sum \varphi_{C}+\sum \varphi_{E}$ with EI(S) (now different from $H(V_{t+1})$) is very strong in the random probabilistic example. $\langle H\left(V_{i,t+1}\right)\rangle$, however, reflects the average degree of noise present in the system in addition to its mechanistic constrains, thus limiting the correlation between $\langle H\left(V_{i,t+1}\right)\rangle$ and $\sum \varphi_{C}\;+\;\sum \varphi_{E}$.

## Appendix B. Practical Measures of Integrated Information and Composition

Attempts to develop practically applicable, empirical measures of integrated information [56,74,75,76,77] are largely based on $\phi_{2.0}$, the version of integrated information proposed in [58]. While the role of composition in accounting for the quality of phenomenal experience had already been recognized then [57], it was not incorporated in the quantitative measure $\phi_{2.0}$. A compositional analysis adds a layer of combinatorial complexity to the already extensive computational demands of evaluating the integrated information of a system. To elucidate the quantitative impact of composition on Φ, in the following, we compare $\Phi_{\subseteq }$ to a state-dependent and state-averaged, non-compositional measure of integrated information. Throughout we denote compositional measures of system-level integrated information by Φ, non-compositional measures by ϕ.

First, we define $\phi_{H}\left(v_{t}\right)$, a non-compositional measure of information integration, which only assesses how Ψ affects the constraints specified by the full set $V_{t}=S$ in state $v_{t}$:

(A1) $$\phi_{H}\left(v_{t}\right)=\min_{\Psi }\left(\min\left(\varphi_{C}^{\Psi }\left(v_{t}\right),\varphi_{E}^{\Psi }\left(v_{t}\right)\right)\right),$$

again using $D_{KL}$ to evaluate $\varphi_{C/E}^{\Psi }\left(v_{t}\right)$, the difference in the cause/effect repertoire of $v_{t}$ before and after the partition Ψ (Equation (16)). $\phi_{H}$ is closely related to $\phi_{2.0}$ [58], and even more so to $\phi_{2.5}$ as defined in [78], since $\phi_{2.0}$ only takes constraints of $v_{t}$ onto the prior states of *S* into account.

Both, $\Phi_{\subseteq }$ and $\phi_{H}$ consider unidirectional spatial partitions (Figure 9b) as introduced with $\Phi_{3.0}$ in [27] to evaluate whether each part of the system specifies intrinsic information about the prior and next state of the rest. In addition, Φ is a state-dependent measure, not a state-independent property of a system. Accordingly, also the partition that makes the least difference, $\Psi^{*}=\mathrm{argmin}\left(\Phi \right)$, should be identified independently for each state. Doing so for both $\Phi_{\subseteq }$ and $\phi_{H}$, we find that for many systems $\phi_{H}=0$ on average, regardless of the average value of $\Phi_{\subseteq }$ (Figure A2a). Overall, $\Phi_{\subseteq }$ and $\phi_{H}$ are only weakly correlated with $\rho_{SP}\left(\Phi_{\subseteq },\phi_{H}\right)=0.24$ for reversible systems (see Figure A2 legend).

This dissociation between $\Phi_{\subseteq }$ and $\phi_{H}$ can be understood based on our example system MCX in state (0,1,1) (Figure 3, Table 1), which specifies a value of $\Phi_{\subseteq }=1.02$ bits, where the minimum is found for $\sum \Delta \varphi_{E}$, under the partition $\Psi^{*}=\left\{\left\{MC_{t},MCX_{t+1}\right\},\left\{X_{t},\emptyset \right\}\right\}$. This partition eliminates $\varphi_{E}\left(X_{t}=1\right)$, $\varphi_{E}\left(CX_{t}=\left(1,1\right)\right)$, and $\varphi_{E}\left(MCX_{t}=\left(0,1,1\right)\right)$, which sums to $\Phi_{\subseteq }=1.02$ bits.

By contrast, $\phi_{H}=0$ bits as the information specified by $MCX_{t}=\left(0,1,1\right)$ about $MCX_{t-1}$ is reducible to the information specified by $MC_{t}=\left(0,1\right)$ alone (Figure 3). The partition $\Psi =\left\{\left\{MC_{t},MCX_{t-1}\right\},\left\{X_{t},\emptyset \right\}\right\}$ does not affect the cause information specified by $MCX_{t}=\left(0,1,1\right))$. Nevertheless, $X_{t}=1$ clearly specifies information about the prior (and next) states of the rest of the system. However, this only becomes apparent when the system’s intrinsic information is evaluated in a compositional manner. Under the same partition that leads to $\phi_{H}=0$ bit, $\sum \Delta \varphi_{C}$ amounts to 2.23 bits.

Generally, $\phi_{H}=0$, whenever the information specified by a part of the system is redundant in information-theoretic (extrinsic) terms, that is, to predict the next system state or to infer the previous system state given the present state of the system. Yet, the system may still be causally integrated, in the sense that every part of the system in its current state specifies causal, intrinsic information about the rest.

Most proposed empirical measures of information integration [56,74,75,76,77] do not evaluate ϕ in a state-dependent manner, but rather as a difference in conditional entropies or mutual information under a (bidirectional) system partition (but see [78], which includes state-dependent measures). For comparison, we define a state-averaged version of ϕ, termed $\phi_{AV}$, in which the same partition Ψ is applied across all system states:

(A2) $$\phi_{AV}=\min_{\Psi }{\langle \varphi_{E}\left(v_{t},\Psi \right)\rangle }_{v_{t}\in \Omega_{S}}.$$

Again, Ψ is a unidirectional partition between sets of system elements. Note that $\phi_{AV}$ basically corresponds to the conditional transfer entropy from one part of the system to another as defined in [56], but imposing a uniform distribution across $V_{t}$ (which is equivalent to the stationary distribution for the set of ER systems). Moreover, $\phi_{AV}$ is defined in terms of $\varphi_{E}$ only, as the *average* causal constraints imposed by one part of the system onto the rest are largely symmetrical. By contrast, for the state-dependent measures $\Phi_{\subseteq }$ and $\phi_{H}$, we evaluate both $\varphi_{C}$ and $\varphi_{E}$, and take the minimum between the two, since the state-dependent cause and effect repertoires capture different conditional probabilities (see Figure 3).

As shown in Figure A2b, we observe a stronger correlation between the state-dependent compositional $\Phi_{\subseteq }$ and the state-averaged measure $\phi_{AV}$ than for $\phi_{H}$. Moreover, $\phi_{AR}=0$ whenever $\Phi_{\subseteq }=0$ and not otherwise. As seen above, $\phi_{H}$ may be zero even if every part of the system is causally connected with the rest of the system. This is because, for each individual system state, there may still be a part of the system that is redundant in information-theoretic terms, i.e., in order to predict the next system state. It is only if the same part is always redundant, that it actually has no causal impact on the rest of the system, in which case both $\phi_{AR}=0$ and $\Phi_{\subseteq }=0$ [27,31,79].

Figure A2b also shows that $\phi_{AR}$ takes on only a few discrete values in the evaluated deterministic systems, corresponding to the minimal average (extrinsic) information “sent” from one part of the system to another from time *t* to t+1. By contrast, $\Phi_{\subseteq }$ is much more widely distributed, identifying differences in the causal composition of the respective systems that would otherwise remain hidden, and that characterize the intrinsic information lost through the system partition.

In all, a non-compositional measure of integrated information may serve as a practical indicator for a system’s *capacity* for Φ, if it is evaluated as an average across (all) possible system states, as done for $\phi_{AR}$. However, in order to assess the amount of integrated information Φ of a system in a particular state, the system’s causal composition cannot be neglected.

Finally, note that the choice of permissible partitions plays a crucial role in determining the value of Φ, and the class of systems for which Φ=0. In line with the canonical measure, $\Phi_{\subseteq }=0$, $\phi_{H}$ = 0, and $\phi_{AV}$ = 0 for any set of elements in which a subset of nodes is connected to the rest in a purely feedforward manner (see Figure 6a,c). Proposed practical measures of integrated information, such as geometric integrated information $\phi_{G}$ [56], decoder-based integrated information $\phi^{*}$ [75], or stochastic interaction (SI) [74,76], typically evaluate bidirectional partitions between sets of system elements, as described for $\phi_{2.0}$ [58] and in [78]. However, unidirectional partitions are necessary to evaluate whether a system specifies integrated information about both its causes and effects (the prior and next state of the system), which is a requirement for being a “whole” from the intrinsic perspective of the system [27].
